# Prevalence and associated factors of depression and posttraumatic stress disorder among trauma patients: multi-centered cross-sectional study

**DOI:** 10.3389/fpsyt.2025.1447232

**Published:** 2025-03-07

**Authors:** Eden Alemayehu Gebresenbet, Samson Zegeye, Tolesa Diriba Biratu

**Affiliations:** ^1^ Medical Doctor and Psychiatrist, Eka Kotebe General Hospital, Addis Ababa, Ethiopia; ^2^ School of Public Health, St. Paul’s Hospital Millennium Medical College, Addis Ababa, Ethiopia

**Keywords:** depression, mental health, post-traumatic stress disorder, trauma, Addis Ababa, Ethiopia

## Abstract

**Background:**

Stress-related disorders, such as post-traumatic stress disorder (PTSD), are expected to be the leading cause of global mortality and morbidity by 2030. However, there is limited information on the prevalence of depression and PTSD among trauma patients in Ethiopia. Our study was aimed at determining the prevalence of depression and PTSD and factors affecting it among trauma patients.

**Methods:**

A hospital-based, multi-centered, cross-sectional study was conducted among 621 patients who visited the trauma outpatient clinic from April to June 2023. The stratified random sampling technique was used to select participants. Data were collected using standardized and pretested structured questionnaires and face-to-face interviews. Post-traumatic stress disorder was measured by the posttraumatic stress disorder checklist for DSM-5 (PCL-5), and depression was assessed by the Patient Health Questionnaire (PHQ-9). Data were analyzed using STATA version 14.1. Bivariable and multivariable logistic regression models were used to examine the association between outcome and independent variables. P-values less than 0.05 were considered statistically significant.

**Results:**

In this study, 621 study participants were involved, with a response rate of 100%. More than half (55%) of the participants were male. The participants’ median age was 32 years, and the interquartile range (IQR) ranged from 25 to 43 years. The prevalence of depression was 35.4% (95% CI: 31.65–39.2), and the prevalence of PTSD was 14.2% (95% CI: 11.4–16.9). In multivariable analysis, being female (AOR = 1.58, 95% CI: 1.05-2.35), having no formal education (AOR = 1.7, 95% CI: 1.01- 2.85), having a low income (AOR = 4.5, 95% CI: 1.93-10.70), and having poor social Support (AOR=2.04, 95% CI 1.34-3.10) and multiple traumatic events (AOR=7.2, 95% CI: 4.1-12.7) were significant predictors of depression. For post-traumatic stress disorder, being female (AOR=2.7, 95% CI 1.46-5.06), no formal education (AOR=2.61, 95% CI: 1.25-5.46), urban residency (AOR=2.11, 95% CI: 1.14-3.90), having depression (AOR=7.01, 95% CI: 3.65-13.46), and multiple traumatic events (AOR=8.08, 95% CI: 2.83-23.14) were the associated factors identified.

**Conclusion and recommendation:**

The study revealed high levels of depression and post- traumatic stress disorder among trauma patients. Targeted interventions addressing socio-demographic disparities, such as income and education levels, alongside psychosocial support, are imperative.

## Introduction

Trauma is a deeply distressing experience that profoundly disrupts an individual’s ability to cope and function. Defined by the Diagnostic and Statistical Manual of Mental Disorders, Fifth Edition (DSM-5) (1), traumatic events involve exposure to actual or threatened death, serious injury, or sexual violence, whether experienced directly, witnessed, or learned about. Unfortunately, these traumatic incidents are all too common in people’s lives, ranging from unexpected deaths to accidents (2, 3).

The impact of trauma-related psychiatric illnesses poses a significant public health concern, affecting individuals worldwide, including those with pre-existing mental and substance use disorders. Extensive research consistently demonstrates a higher prevalence of mental illness in populations exposed to traumatic events. Various literatures confirm the greater prevalence of mental illness in populations exposed to traumatic events (4–7).

Trauma exposure is a significant risk factor for developing depression and post-traumatic stress disorder (PTSD). A systematic review and meta-analysis conducted on adult survivors of wars in conflict-affected countries between 1989 and 2019 revealed a point prevalence of 26.51% for PTSD and 23.31% for major depression. These findings indicate that in 2019, approximately 316 million adult war survivors worldwide suffered from PTSD and/or major depression, with a significant burden in low- or middle-income countries (8).

Globally, around 13 million people develop PTSD each year (9). The World Mental Health Survey reported a lifetime prevalence of PTSD of 3.9% in the general population and 5.6% in trauma-exposed individuals (10) and is frequently encountered in primary care settings, particularly among patients with comorbid medical conditions (11, 12). In Africa, the pooled prevalence of probable PTSD across studies was found to be 22%, with higher estimates in war-exposed regions (13). Additionally, a systematic review and meta-analysis conducted in Ethiopia revealed a pooled prevalence of post-traumatic stress disorder of 39.28% (14).

PTSD is associated with significant disability, accounting for approximately 0.6% of the global years lived with disability (15). Individuals with PTSD experience impaired role functioning, reduced life opportunities, and substantial economic costs (16). Work impairment associated with PTSD results in an annual lost productivity cost of over $3 billion in the United States alone (17). Moreover, PTSD increases the risks and consequences of comorbid medical conditions, such as acute coronary syndrome and diabetes (18, 19).

Depression is another prevalent mental illness among trauma patients, affecting approximately 280million globally (20), and holds the highest lifetime prevalence among psychiatric disorders, ranging from 5% to 17%, with higher prevalence in developing nations (17, 21). In Africa, about 29.19 million people suffer from depression, and in Ethiopia, depression contributes to about 6.5% of the burden of diseases (13, 22).

Depression can worsen the prognosis and course of various medical conditions, including cardiovascular diseases, diabetes, and HIV/AIDS (12). It is associated with increased disability, reduced quality of life, and higher mortality rates (23). Depression also incurs significant economic costs, including healthcare expenditures and lost productivity (24–26).

While PTSD and depression are prevalent mental health conditions globally, their prevalence and access to treatment can significantly vary between low-income and high-income countries. Individuals in low-income countries are particularly vulnerable to trauma due to factors such as violence, natural disasters, and poverty, increasing their risk of developing PTSD and depression (10). Low-income countries bear a heavier burden of these disorders, with over 80% of affected patients residing in these regions, where access to treatment is limited (11). Furthermore, there is a strong comorbidity between PTSD and depression (11, 12).

Previous community studies conducted in trauma-affected populations in Ethiopia have reported varying prevalence rates of PTSD (ranging from 17% to 59.8%) (11–14) and depression (ranging from 7% to 81%) (27–29). Despite the high prevalence and significant impact of PTSD and depression, there is a considerable treatment gap in low-income countries, including Ethiopia (15). The majority of individuals with these disorders do not receive appropriate mental health care. Barriers to accessing mental health services in Ethiopia include limited availability of trained professionals, inadequate infrastructure, stigma and discrimination, and lack of awareness.

Given the limited data on the prevalence and impact of PTSD and depression in trauma patients attending outpatient clinics in Ethiopia, conducting a study to assess the burden of these disorders and identify associated factors is crucial. Studying trauma patients in Ethiopia provides valuable insights into how limited awareness of mental health, a constrained healthcare system, and socioeconomic factors affect their care. This information can inform the development of targeted interventions and improve the provision of mental health care for individuals affected by trauma.

## Materials and methods

### Study setting and study period

A hospital-based cross-sectional study was conducted from April to June 2023 to assess the prevalence of depression and PTSD and its associated factors among trauma patients on follow-up at six trauma clinics of specialized teaching hospitals in Addis Ababa, Ethiopia. Addis Ababa is the capital city of Ethiopia and is located at 2355 meters above sea level. Based on the population projection of the Central Statistical Agency of Ethiopia in 2021, Addis Ababa has a total population of 3,774,000, of whom 1,782,000 are men (30).

In this study, a stratified random sampling approach was used to select six hospitals from 52 in Addis Ababa, based on high patient burden and trauma flow. The selected hospitals include Africa Leprosy Rehabilitation and Training Center, Yekatit 12 Medical College, Addis Ababa Burn Emergency Trauma Hospital, and Zewditu Memorial Hospital (trauma centers), along with Tikur Anbessa Specialized Hospital and St. Paul’s Hospital Millennium Medical College (referral hospitals). Tirunesh Beijing Hospital was used for the pretest study. Collectively, these six hospitals monitor approximately 37,000 trauma patients in follow-up care.

### Participant, sample size, and sampling

The source population was all patients attending selected hospitals’ trauma clinics. Selected patients visiting the selected hospitals’ trauma outpatient clinics during the study period and fulfilling the inclusion criteria were included in the study population. Patients who were in critical health condition were excluded from the study. This exclusion was necessary as these individuals may be undergoing significant medical interventions, which can impair their mental state and ability to provide informed consent. Additionally, patients who were unable to respond to the questionnaire and refused to participate were also excluded from this study.

The sample size was calculated using a single population proportion formula by considering the following assumptions: Prevalence of PTSD and depression: 59.8% and 24.5%, respectively (31, 32). The margin of error of 5%, with a 95% confidence level and an adjustment of a 10% non-response rate, was used. The final sample size calculated by adding 10% for the non-response rate was 621.The stratified random sampling technique was employed, and the sample size for each strata (hospital) was allocated proportionally based on the total number of trauma patients on follow-up, and a simple random sampling technique was used to select participants visiting the selected hospitals’ outpatient trauma clinics using the lottery method technique.

### Study variables

The dependent variable is the presence of depression and PTSD dichotomized as “YES” and “NO”. The independent variables in this study were divided into three categories. They were a) sociodemographic—this category included variables such as age, gender, residence, marital status, educational status, and employment status, and b) trauma-related factors—this category included variables such as the type of trauma and frequency of trauma exposure. C) Social support-related factors.

### Operational definitions

- Traumatic event: Exposure to actual or threatened death, serious injury, or sexual violence in one or more of four ways: (i) experiencing the event directly; (ii) witnessing events occurring to a person; (iii) being informed that such a traumatic event has happened to one’s family member or a friend; (iv) experiencing repeated or extreme exposure and the details of its adversities, such as reminders of the first response (1).
- Traumatic or stressful events include being a witness to or being involved in a violent accident or crime, military combat, or assault; being kidnapped; being involved in a natural disaster; being diagnosed with a life-threatening illness; or experiencing systematic physical or sexual abuse (12).
- A mental illness is characterized by a clinically significant disturbance in an individual’s cognition, emotional regulation, or behavior. It is usually associated with distress or impairment in important areas of functioning (33, 34).
- Depression: The patient must fulfill the defining criteria for depression according to PHQ-9, i.e., a score of 5 or greater has depression, and a score less than 5 has no depression. Therefore, a score of 5 and above is categorized as having depression and coded as “(1).” and the others who scored less than 5 coded as “0” (35).
- Post-Traumatic Stress Disorder (PTSD): The patient must fulfill the defining criteria for PTSD according to PCL-5. Therefore, a score of 31 and above is categorized as having PTSD and coded as “(1).” and the others who scored less than 31 coded as “0” (36, 37).
- Social support—OSLO-3 scale: an individual is deemed to have poor social support if he or she scores 3-8, moderate social support if he or she scores 9-11, and good social support if he or she scores 12-. Therefore, a score of 12 and above is categorized as having good social support and coded as “(2),” while a score of 9-11 is categorized as having moderate social support and coded as “(1),” and the others who scored between 3-8 coded as “0” (38).

### Data collection methods and procedures

A structured standard questionnaire was used by adapting from LEC-5 (39), PCL-5 (37), PHQ-9 (35), and the OSLO-3 scale (38). The questionnaire was designed to obtain information on the socio- demographic characteristics of participants.

The questionnaire consisted of five sections. The first section contains the socio-demographic characteristics. Such as age, gender, residence, marital status, educational status, employment, and monthly income, the second section was about social support (38), while the third section aimed to identify and assess a participant’s trauma history (39). The fourth section was dedicated to assessing depression (35), while the fifth and final section centered on post-traumatic stress disorder (PTSD) (37). Previous research has demonstrated the PCL-5’s high internal consistency reliability in Ethiopia, with Cronbach’s alpha coefficients above 0.85 (40). Our own study found similarly strong reliability, with a Cronbach’s alpha of 0.90 for the PCL-5.

To assess depression, the study employed the PHQ-9 questionnaire, comprising nine items that were scored on a scale of 0 (“not present”) to 3 (“nearly every day”). The total score for the PHQ-9 fell within the range of 0 to 27. The PHQ-9 measure used in our study has also been validated for use in the Ethiopian context, evidenced by a Cronbach’s alpha of 0.95 and significant correlations with established depression measures (35). To further ensure cultural appropriateness, we conducted a pilot study with participants from the target population, which provided additional support for the suitability of these measures.

The administration of the questionnaires was carried out through face-to-face interviews conducted by two skilled nurses, both of whom possessed bachelor’s degrees and had undergone rigorous training. The principal investigator provided supervision throughout the process. Individual interviews were conducted with trauma patients within hospital settings, ensuring a personalized approach. The completion of the questionnaires typically took approximately 20 to 30 minutes, allowing for thorough data collection. The principal investigator diligently reviewed the collected data on a daily basis, meticulously verifying its completeness and consistency. Any individual records found to contain incomplete data were excluded from the subsequent analysis, ensuring the integrity and reliability of the study findings.

### Data processing and analysis

The collected data were promptly cleaned and entered directly using the Kobo Collect version 5 and then exported to STATA version 14 for further analysis. STATA 14.1 is a robust statistical software package that provides comprehensive analysis tools, user-friendly data management capabilities, and features that enhance reproducibility through scripting. Additionally, it offers extensive documentation and strong graphical capabilities, making it particularly well-suited for analyzing the prevalence of depression and PTSD among trauma patients.

Prior to statistical analysis, a rigorous data cleaning process was conducted to identify and address outliers and inconsistencies within the dataset. Descriptive statistics were utilized to gain a comprehensive overview of the data. Tables and graphs were employed to present the results of descriptive statistics for categorical variables, while measures such as mean and standard deviation were used for normally distributed continuous variables. For non-normally distributed continuous variables, the median and interquartile range (IQR) were employed as summary measures. Data were analyzed using a bivariate and multivariable logistic regression model in order to identify the association between the independent and the outcome variables. P-values less than 0.05 were considered statistically significant.

## Results

### Socio-demographic characteristics of study participants

In this study, a total of 621 participants were involved, with a 100% response rate. Of the participants, more than half, 351 (56%), were male. The median age of the participants was 32 years, with an interquartile range (IQR) of 25-43 years. Nearly half, 300 (48.3%), of the participants were single, while more than one-third, 252 (40.6%), had secondary education. The majority, 478 (77%), were employed, with more than one-fourth, 174 (28.5%), working as government employees. The median monthly income was 4,700 birrs, with an interquartile range of 2,000-9,000 birrs. Most participants (376, 60.6%) were from rural areas (**Table 1**).

**Table 1:** Socio-demographic characteristics of the study participants at selected public hospitals in Addis Ababa, July 2023.

Variable	Category	Frequency (N=621)	Percentage (%)
Age	18-25	170	27.4
	26-35	178	28.7
	36-60	259	41.7
	>60	14	2.2
	Median (IQR)	32 (23-43)	
Gender	Male	351	56.5
	Female	270	43.5
Residency	Rural	376	60.6
	Urban	245	39.4
Education	No formal education	144	23.2
	Read and write	52	8.4
	Primary	150	24.1
	Secondary	252	40.6
	Tertiary	23	3.7
Marital status	Unmarried	300	48.3
	Married	286	46.05
	Divorced	19	3.1
	Widowed	16	2.6
Employment	Yes	478	77
	No	143	23
Occupation	Housewife	32	5.1
	Merchant	125	22.4
	Government	174	28.5
	Private	115	18.5
	Daily laboror	35	6
	Farmer	29	4.8
	No occupation	111	14.7
Monthly Income	0-2000 ETB	175	28.2
	2001-5,000	202	32.5
	5001-9,000	100	16
	>9,000	144	23.2
	Median (IQR)	4,700 (2000-9,000)	

### Social support characteristics

**Figure 1:**
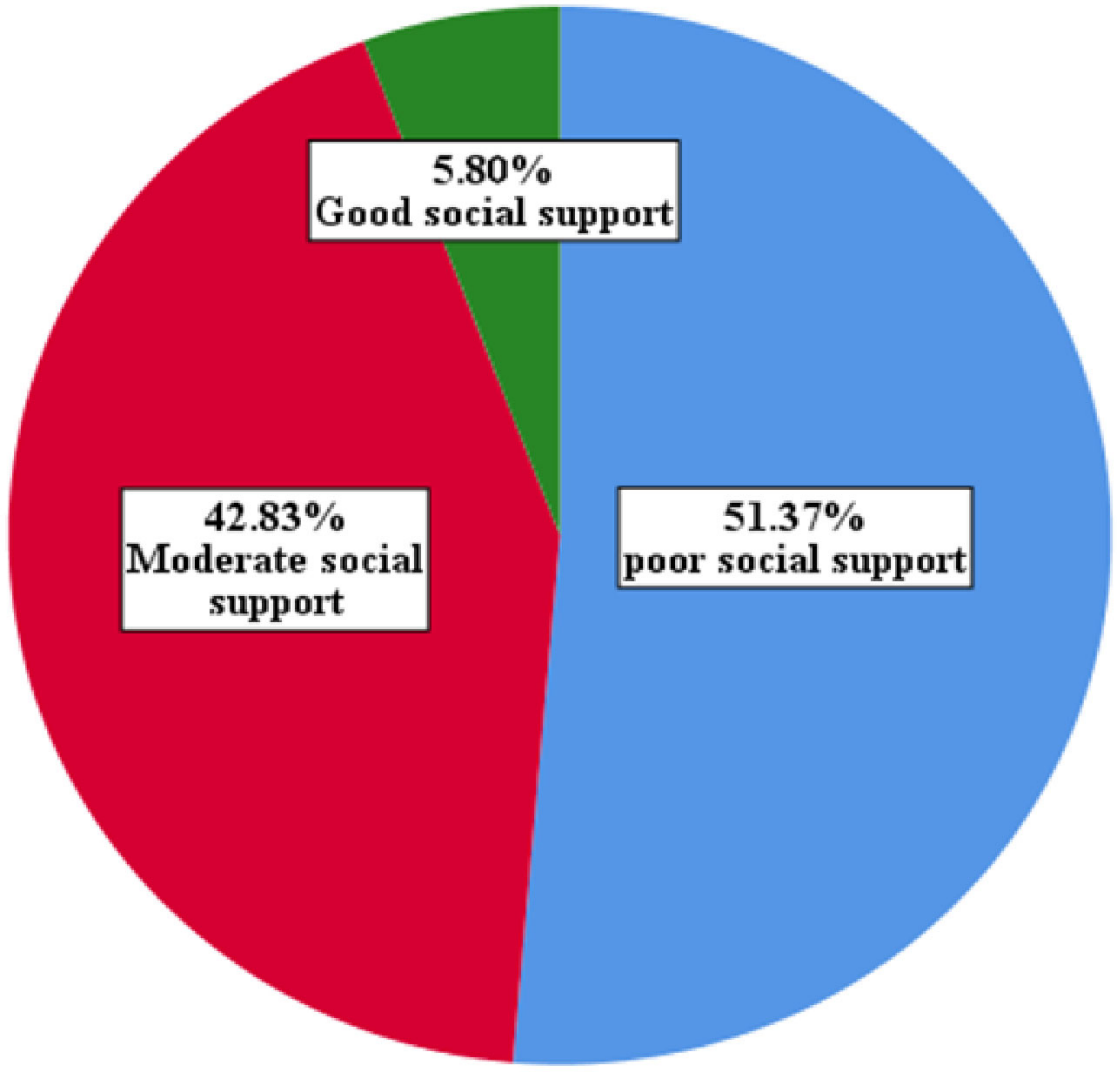
Social support characteristics of the study participants at selected public hospitals in Addis Ababa, July 2023.

As shown in **Figure 1**, among the participants, almost half of 319 participants (51.4%) reported having a poor social support system, while 266 participants (42.83%) reported having a moderate level of social support. A small number of participants, 36 (5.8%), indicated that they have a strong social support system.

### Trauma-related characteristics

#### Types of traumas

**Figure 2:**
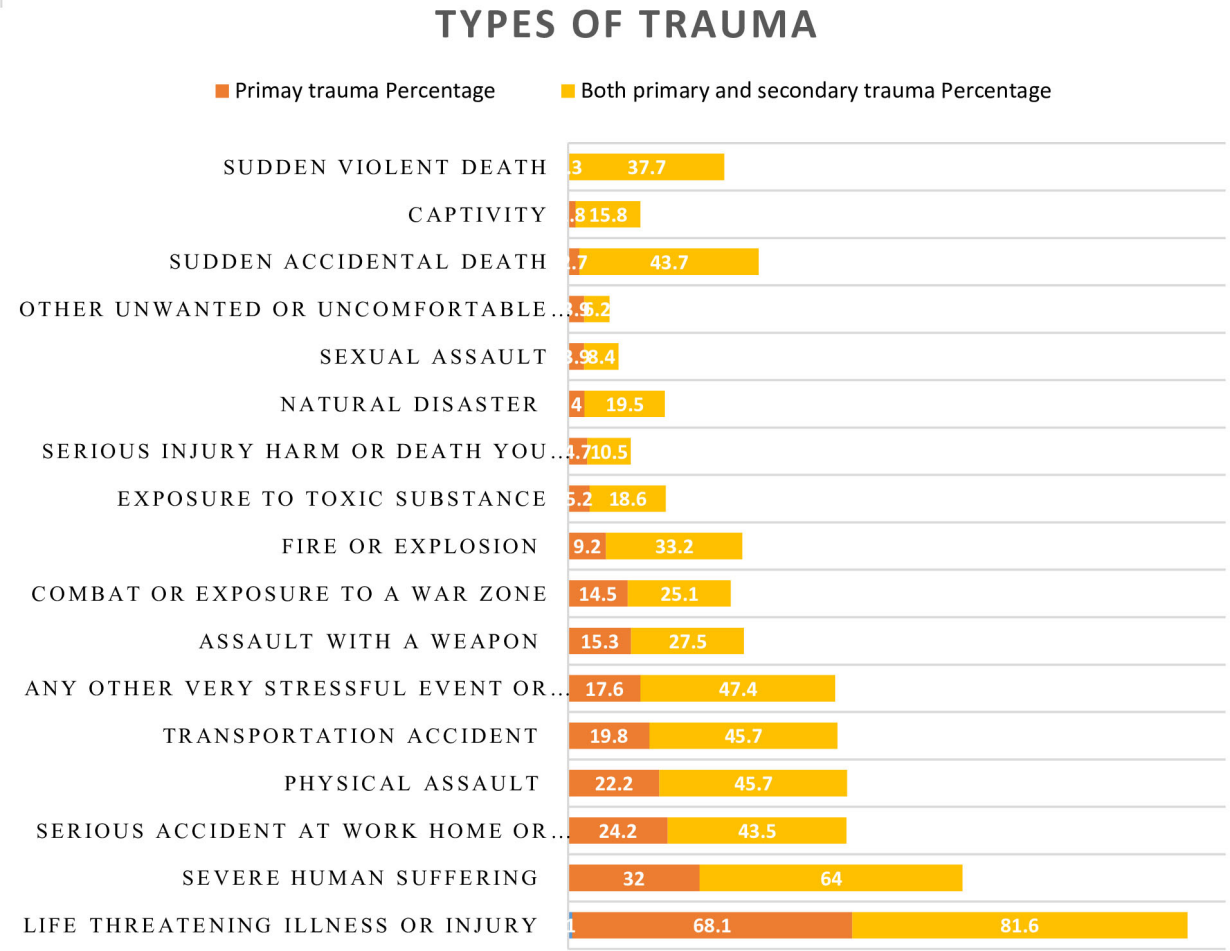
Types of Trauma reported by the study participants at selected public hospitals in Addis Ababa, July 2023.

One participant (0.2%) reported all forms of traumatic experiences assessed. The most prevalent traumatic event was life-threatening illness or injury (507, 81.6%), followed by severe human suffering (64%), any other very stressful event or experience (47.4%), car accidents, and physical assaults (45.7% each) (**Figure 2**).

#### Frequency of trauma exposure

**Figure 3:**
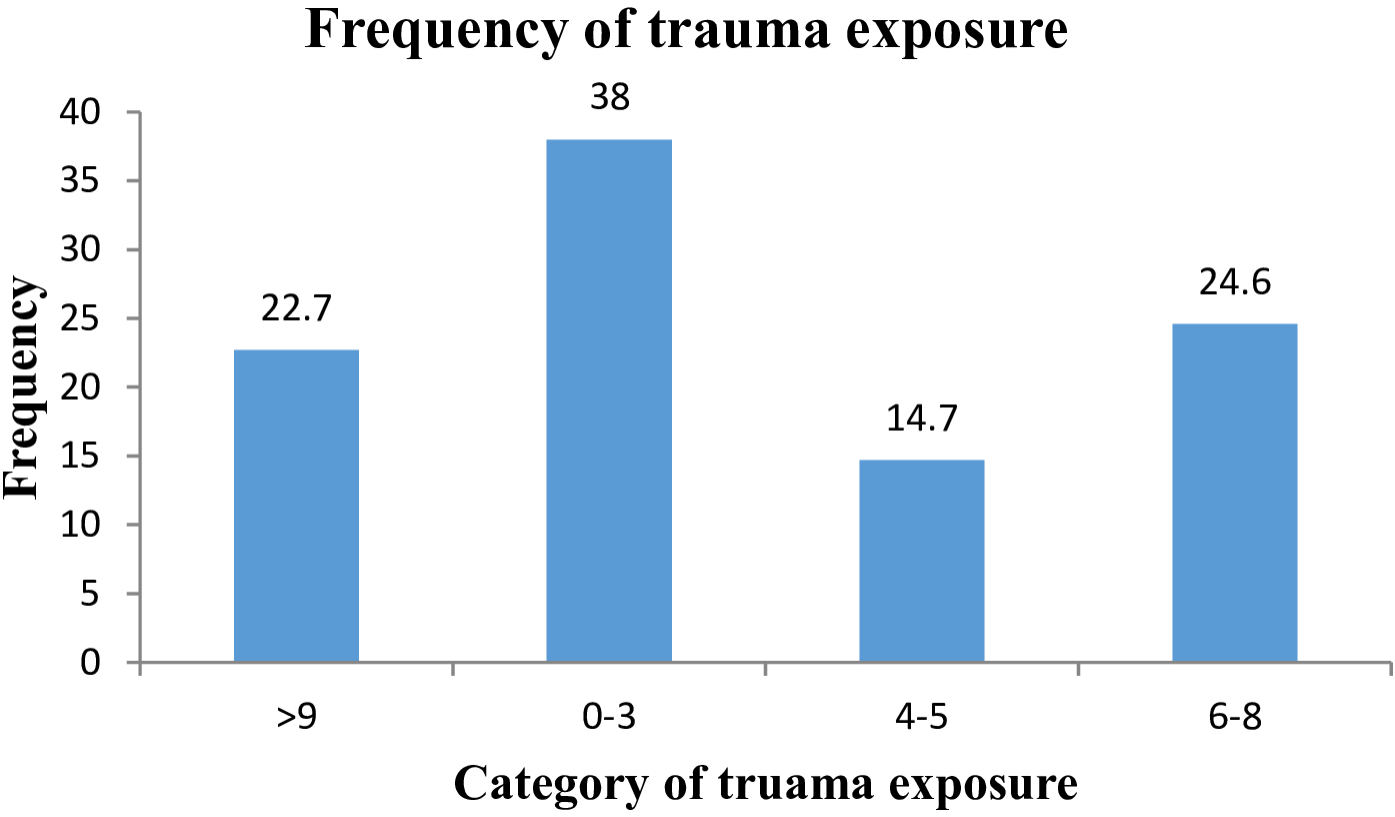
Frequency of trauma exposure reported by the study participants at selected public hospitals in Addis Ababa, July 2023.

The study found that the median number of traumatic experiences reported by participants was 5, with an interquartile range of 3-8. A total of 236 (38%) of participants reported experiencing 1-3 traumatic events, while 153 (24.6%) indicated that they had experienced 6-8 traumatic events. A further 149 (22.7%) of participants reported experiencing more than 9 traumatic events (**Figure 3**).

### Prevalence of depression and PTSD

**Figure 4:**
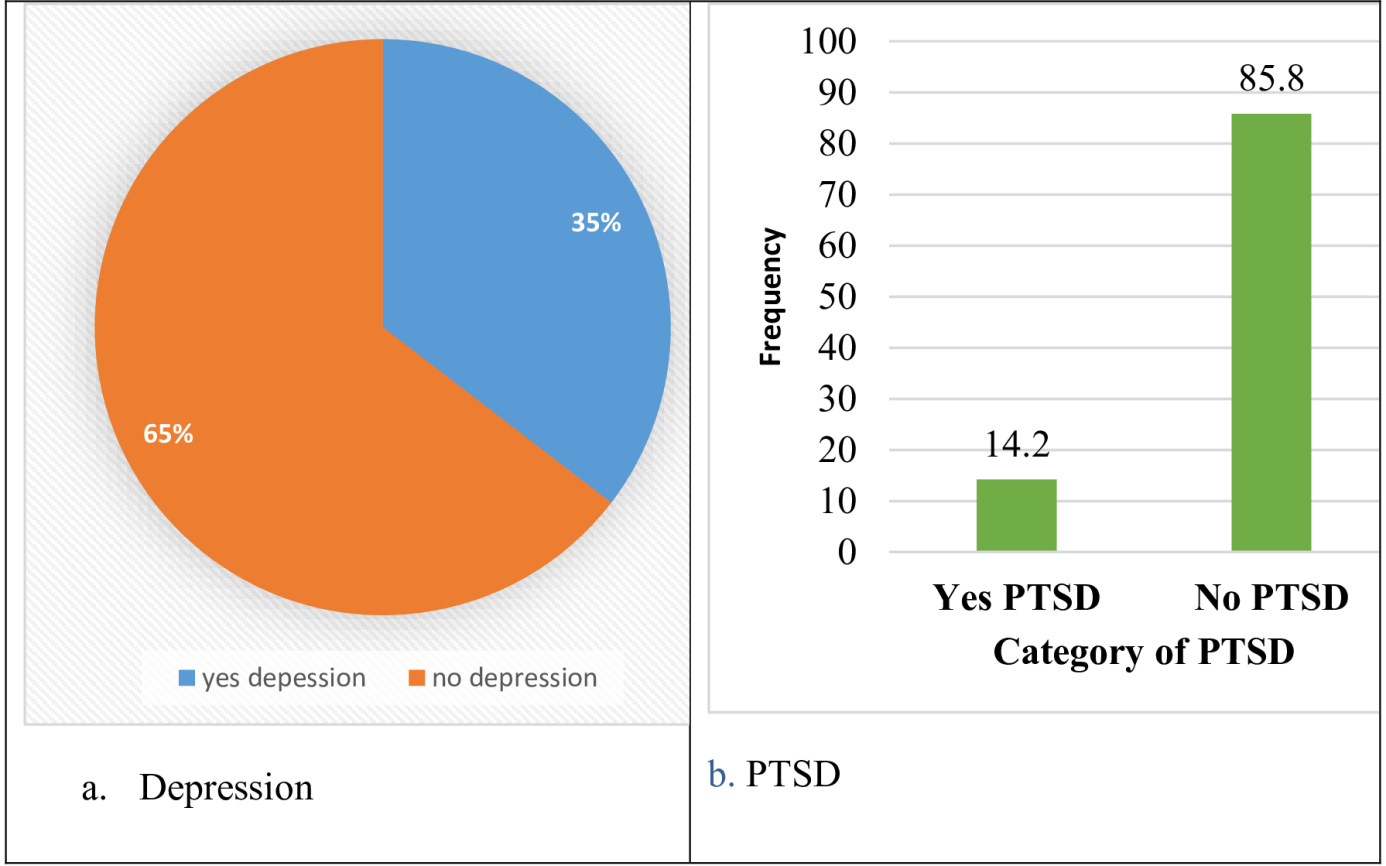
.

As shown in **Figure 4**, the estimated prevalence of depression was 35.4%, with a 95% CI of 31.65%– 39.2%, with 220 participants screening positive for depression. Similarly, the estimated prevalence of PTSD was 14.2% with a 95% CI of 11.4%–16.9%, with 88 participants screening positive for PTSD (**Figure 4**).

### Factors associated with depression

This study employed multivariable logistic regression to identify significant predictors of depression among trauma patients. Variables with a p-value of less than 0.25 in the bivariable analysis were included in the multivariable analysis to determine the independent variables that could predict depression. The final parsimonious model was built using the backward stepwise model-building technique. The bivariable analysis identified age, gender, educational status, employment, income, social support, exposure to multiple traumatic events, and posttraumatic stress disorder as candidate variables for the multivariable analysis. The results of the multivariable analysis indicated that female gender, low income, unemployment, poor social support, lack of formal education, and exposure to multiple traumatic events were significant predictors of depression. No significant differences in depression symptoms were found with respect to age, marital status, or posttraumatic stress disorder (**Table 2**).

**Table 2:** Bivariable and multivariable logistic regression analysis results of depression among traumatic patients at selected hospitals, Addis Ababa, Ethiopia, 2023 (N = 621).

Variable	Category	Depression N (%)		COR (95CI)	AOR (95 CI)	p-value
		Yes	No			
Age	18-25	70 (11.3)	100 (16.1)	1.17 (0.79-1.74)	1.35 (0.71-2.56)	0.362
	26-35	48 (7.7)	130 (20.9)	0.62 (0.41-0.93)	0.66 (0.37-1.18)	0.166
	>36	102 (16.4)	171 (27.5)	Ref.	Ref.	
Gender	Men	102 (16.4)	249 (40.1)	Ref.	Ref.	
	Women	118 (19)	152 (24.5)	1.89 (1.36-2.64)	1.58 (1.05-2.35)	0.025*
Residence	Rural	129 (20.8)	247 (39.77)	0.88 (0.63-1.23)	1.67 (1.07-2.59)	0.021*
	Urban	91 (14.65)	154 (24.8)	Ref.	Ref.	
Education	No formal education	64 (10.3)	80 (12.9)	1.85 (1.22-2.81)	1.7 (1.01-2.85)	0.044*
	Only read and write	23 (3.7)	29 (4.7)	1.83 (1.00-3.34)	1.54 (0.77-3.08)	
	Primary	50 (8.05)	100 (16.1)	1.16 (0.76-1.77)	0.98 (0.58-1.64)	
	Secondary and above	83 (13.4)	192 (30.9)	Ref.	Ref.	
Marital status	Married	95 (15.3)	191 (30.8)	Ref.	Ref.	
	Unmarried	109 (17.6)	191 (30.8)	1.15 (0.82-1.61)	0.84 (0.48-1.46)	0.538
	Divorced/widowed	16 (2.6)	19 (3.1)	1.69 (0.83-3.44)	1.12 (0.45-2.76)	0.804
Employment	No	78 (12.56)	65 (10.5)	2.84 (1.94-4.16)	2.36 (1.14-4.87)	0.020*
	Yes	142 (22.9)	336 (54.1)	Ref.	Ref.	
Monthly Income (Eth Birr)	0-2,000	89 (14.3)	86 (13.85)	9.61 (5.12-17.96)	4.55 (1.93-10.70)	0.001*
	2,001-5,000	77 (12.4)	125 (20.1)	5.72 (3.07-10.63)	5.42 (2.53-11.61)	0.000*
	5001-9,000	40 (6.4)	60 (9.66)	6.19 (3.13-12.23)	3.26 (1.65-6.43)	0.001*
	>9,000	14 (2.25)	130 (20.9)	Ref.	Ref.	
Social support	Poor social support	147 (23.7)	172 (27.7)	2.68 (1.90-3.78)	2.04 (1.34-3.10)	0.001*
	Moderate and strong social support	73 (11.8)	229 (36.9)	Ref.	Ref.	
Frequency of trauma exposure	0-3	42 (6.76)	194 (31.24)	Ref.	Ref.	
	4-5	28 (4.51)	63 (10.14)	2.05 (1.18-3.58)	1.79 (0.97-3.49)	0.069
	6-8	63 (10.14)	90 (14.5)	3.23 (2.03-5.14)	4.02 (2.31-6.98)	0.000*
	>9	87 (14.01)	54 (8.7)	7.44 (4.62-11.97)	7.24 (4.10-12.79)	0.000*

*Statistically significant, p <0.05.

After adjusting for other variables, the odds of depression were 42% higher among female participants than their male counterparts (AOR=1.58; 95% CI: 1.05, 2.35). Participants who had no formal education exhibited 30% higher odds of experiencing depression compared to individuals with an education level above secondary school (AOR=1.7; 95% CI: 1.01, 2.85). The odds of having depression among unemployed participants were 2.36 times higher than among employed participants (AOR = 2.36; 95% CI: 1.14, 4.87). In addition, participants with a monthly income of birrs 0-2000, 2001-5000, and 5001-9000 had 4.55, 5.42, and 3.26 times higher odds of depression, respectively, compared to those with a monthly income above 9,000 birr (AOR=4.55; 95% CI: 1.93, 10.70), (AOR=5.42; 95% CI: 2.53, 11.61), and (AOR=3.26; 95% CI: 1.65, 6.43).

Poor social support was also found to be a significant predictor of depression in the study. Participants reporting poor social support had 2.04 times higher odds of depression than those reporting moderate and strong social support (AOR = 2.04; 95% CI: 1.34, 3.10).

Finally, the odds of depression among participants who had experienced more than six traumatic events were four times greater than those who had experienced between zero and three traumatic events (AOR = 4.02; 95% CI: 2.31, 6.98). The odds of depression among participants who had experienced more than nine traumatic events were seven times greater than those who had experienced between zero and three traumatic events (AOR = 7.24; 95% CI: 4.10, 12.79).The study found no evidence of multicollinearity among the independent variables, as evidenced by the Variance Inflation Factor (VIF) values, which were all below 10, with an overall mean VIF of 2.08 and tolerance values greater than 0.1. In addition, the study assessed the goodness of fit of the multivariable logistic regression model using the Hosmer- Lemeshow test (χ² (8)=6.21, p-value=0.55). The test revealed that the model had a good fit. The study also evaluated the discrimination ability of the model using the area under the receiver operating characteristic (ROC) curve, which was found to be 0.80, indicating good discrimination.

### Factors associated with PTSD

After conducting bivariable analysis, variables with p-values less than 0.25 were included in the multivariable logistic regression to determine the independent variables that could predict PTSD. The final parsimonious model was built using the backward stepwise model-building technique. From the bivariable analysis, it was seen that the independent factors such as age, gender, educational status, residence, employment, income, social support, exposure to multiple traumatic events, and depression were candidate variables for multivariable analysis with a p-value < 0.25.The multivariable analysis revealed that several significant factors were associated with an increased likelihood of PTSD among trauma patients, including female gender, urban residence, no formal education, poor social support, exposure to multiple traumatic events, and depression (**Table 3**).

**Table 3:** Bivariable and multivariable logistic regression analysis results of PTSD among traumatic patients at selected hospitals, Addis Ababa, Ethiopia, 2023 (n = 621).

Variable	Category	PTSD n (%)		COR (95%CI)	AOR (95%CI)	P-value
		Yes	No			
Age	18-25	30 (4.8)	140 (22.5)	1.17 (0.79-1.74)	1.32 (0.55-3.19)	0.529
	26-35	16 (2.6)	162 (26.1)	0.54 (0.29-0.999)	0.76 (0.31-1.84)	0.545
	>36	42 (6.8)	231 (37.2)	Ref.	Ref.	
Gender	Men	35 (5.6)	316 (50.9)	Ref.	Ref.	
	Women	53 (8.5)	217 (34.9)	2.2 (1.39-3.49)	2.7 (1.46-5.06)	0.044*
Residence	Rural	33 (5.3)	343 (55.2)	Ref.		
	Urban	55 (8.8)	190 (30.6)	3 (1.88-4.79)	2.11 (1.14-3.90)	0.025
Education	No formal Education	31 (4.9)	113 (18.2)	2.63 (1.49-4.63)	2.61 (1.25-5.46)	0.011*
	Only read and write	9 (1.5)	43 (6.9)	2.00 (0.88-4.57)	1.67 (0.61-4.57)	0.314
	Primary	22 (3.5)	128 (20.6)	1.64 (0.89-3.02)	1.54 (0.72-3.29)	0.261
	Secondary and above	26 (4.19)	249 (40.1)	Ref.	Ref.	
Marital status	Married	42 (6.8)	244 (39.3)	Ref.	Ref.	
	Unmarried	42 (6.8)	258 (41.6)	0.95 (0.59-1.50)	0.82 (0.44-1.52)	0.530
	Divorced/widowed	4 (0.6)	31 (5)	0.75 (0.25-2.23)	0.32 (0.08-1.16)	0.083
Employment	No	29 (4.7)	114 (18.4)	1.81 (1.11-2.95)	1.62 (0.62-4.23)	0.324
	Yes	59 (9.5)	419 (67.5)	Ref.	Ref.	
Monthly income (Eth Birr)	0-2,000	38 (6.12)	137 (22.06)	13.04 (3.93-43.23)	3.54 (0.94-13.24)	0.061
	2,001- 5,000	34 (5.48)	168 (27.05)	9.51 (2.86-31.63)	1.67 (0.44-6.38)	0.451
	5,001-9,000	13 (2.09)	87 (14.01)	7.02 (1.95-25.35)	1.30 (0.29-5.69	0.727
	>9,000	3 (0.48)	141 (22.71)	Ref.	Ref.	
Social support	Poor social support	80 (12.9)	239 (38.5)	12.3 (5.83-25.95)	7.46 (3.31-16.84)	0.000*
	Moderate and strong social support	8 (1.29)	294 (47.34)	Ref.	Ref.	
Frequency of trauma exposure	0-3	5 (0.81)	231 (37.2)	Ref.	Ref.	
	4-5	7 (1.13)	84 (13.53)	3.85 (1.18-12.46)	2.59 (0.73-9.18)	0.139
	6-8	29 (4.67)	124 (19.9)	10.80 (4.08-28.6)	4.35 (1.48-12.86)	0.008*
	>9	47 (7.57)	94 (15.1)	23.1 (8.91-59.88)	8.08 (2.83-23.14)	0.000*
Depression	No	15 (30.8)	386 (5.1)	Ref.	Ref.	
	Yes	73 (56.1)	147 (9.3)	12.77 (7.10-22.98)	7.01 (3.65-13.46)	0.000*

*Statistically significant, p <0.05.

After controlling for other variables, female participants had nearly three times higher odds of developing PTSD compared to males (AOR=2.7; 95% CI: 1.46, 5.06). Similarly, participants residing in urban areas had 2.1 times greater odds of PTSD than those in rural areas (AOR=2.11; 95% CI: 1.14-3.90). In addition, the odds of having PTSD among participants who had no formal education were 2.61 times higher than those who had above secondary education (AOR=2.61; 95% CI: 1.25, 5.46).

Social support was also found to be a significant predictor of PTSD, with participants reporting poor social support had seven times higher odds of having PTSD than those who had moderate and strong social support (AOR = 7.46; 95% CI: 3.31, 16.84).

The frequency of trauma exposure experienced was a significant predictor of PTSD as well, with participants who had experienced more than six traumatic events having four times higher odds of PTSD than those who had experienced between zero and three traumatic events and participants who had experienced more than nine traumatic events having eight times higher odds of PTSD than those who had experienced between zero and three traumatic events (AOR = 4.35; 95% CI: 1.48, 12.86) and (AOR = 8.08; 95% CI: 2.83, 23.14), respectively. Additionally, participants with depression had over seven times higher odds of developing PTSD compared to those without depression (AOR=7.01; 95% CI: 3.65, 13.46).

There was no evidence of multicollinearity among the independent variables, as evidenced by the Variance Inflation Factor (VIF) values, which were all below 10, with an overall mean VIF of 1.6 and tolerance values greater than 0.1. The study also assessed the model fit and discrimination ability of the multivariable logistic regression model. The Hosmer-Lemeshow goodness-of-fit test indicated a good fit for the model *(χ²(8) = 9.97, p-value = 0.55).* Additionally, the area under The receiver operating characteristic (ROC) curve was found to be 0.90, indicating good discrimination of the model. These findings provide valuable insights into the risk factor that are associated with PTSD in trauma patients, which can inform the development of effective prevention and intervention strategies.

## Discussion

### Depression

The impact of trauma on mental health has been a topic of interest in many studies, and this research aimed to investigate the prevalence and risk factors of depression among trauma patients. The study found that the estimated prevalence of depression in trauma patients was 35.4%, which is consistent with previous research (42–44). The prevalence was lower than in studies conducted in Somalia and South Sudan (36, 41). Differences in depression prevalence rates may have been partly due to differences in the instrument used to diagnose depression and the characteristics of the study participants. Contrarily, the estimated prevalence of the current study was higher than the studies carried out in Japan and Sri Lanka, 2.6% and 5% for depression, respectively (45, 46); these discrepancies may be due to differences in methodology and population characteristics.

In this study, there was no significant association observed between age and the presence of depression among trauma patients, which aligns with earlier studies conducted in South Ethiopia and Nepal (42, 47). But a study conducted in Somalia reported an association between age and depression (43), which may be attributed to methodological differences.

The study result showed that women had a greater risk of developing depression than men, consistent with previous studies done in the USA, India, Nepal, and Ethiopia (41, 42, 48, 49). This may be due to the influence of sex hormones on depression, leading to more emotional and ruminative reactions to stress in women (12, 22). There is a higher prevalence of depression in females compared to males in the general population as well. The universal higher female-to-male prevalence ratios of depression suggest a primarily biological influence rather than confounding social and economic factors (12).

The study also noted that the lower education levels were associated with a higher risk of depression, which aligns with findings from previous studies (40, 50–52). This may be due in part to the fact that lower levels of education are often associated with lower socioeconomic status, which can increase the likelihood of experiencing traumatic events and make it more difficult to access effective treatment for depression. However, other studies have suggested that higher Education levels may also increase the risk of mental illness, possibly due to greater expectations for success and achievement (41).

In this study, there was no significant association between marital status and the presence of depression among trauma patients, consistent with studies done in Nepal, Somalia, and Ethiopia (36, 41, 53). However, this finding contrasts with studies conducted in the United Kingdom (54) and India (55), possibly due to variations in sample size, methodology, and population characteristics.

In this study, unemployment has a significant association with depression, consistent with the findings in previous studies (36, 46, 56). The study also found that participants in the low-income category had a higher likelihood of experiencing depression compared to those with a high income, supported by previous studies (41, 42, 53). The possible reason might be due to easier access to private healthcare and social support in the early stages of trauma among individuals with higher income.

The odds of developing depression among those who have poor social support were higher when compared to those with strong and moderate social support. This finding suggests that positive social support enhances individuals’ coping capacity, whereas poor social support exacerbates the negative psychological effects of trauma (40–42, 57).

Finally, the study showed a dose–response relationship between trauma exposure and depression, with greater exposure to multiple types of traumatic events predicting greater depression risk. The number of traumatic exposures was a significant predictor of depression, which is consistent with findings from other studies (36, 42, 47). This could be because when an individual is exposed to several traumatic events, they have a greater negative influence on mental health than a single, discrete trauma.

This comprehensive study highlights the prevalence and risk factors of depression among trauma patients. It emphasizes the importance of considering various factors such as gender, education, employment, income, social support, and the cumulative effect of trauma exposure on mental health outcomes. Understanding these factors can contribute to the development of effective interventions and support systems for individuals who have experienced trauma.

### Post-traumatic stress disorder

The impact of trauma on mental health has been researched, and this study aimed to investigate the prevalence and risk factors of PTSD among trauma patients. The lifetime incidence of PTSD is predicted to be 9–15% (12), and the estimated prevalence of PTSD in trauma patients in this study was found to be 14.2%, which is consistent with previous studies conducted in Nepal and Ethiopia (41, 57, 58).

The prevalence was lower than in studies conducted in South Sudan, Northwest Ethiopia, and South Ethiopia (12, 32, 47, 50). These differences might be attributed to variations in the use of measurement tools and cut-off points for diagnosing PTSD, the exposure to multiple traumatic events, differences in study design, and the type and severity of the accidents examined. Numerous studies have shown that the prevalence of PTSD increases along with the level of exposure to traumatic events, such as the quantity or intensity of experienced events (12, 32).

Contrarily, the estimated prevalence of PTSD in this study was higher than the studies conducted in China and Nigeria (59, 60). This variation may be attributed to differences in the assessment instruments used. Regarding age, this study did not find a significant association between age and the presence of PTSD among trauma patients, which aligns with previous studies conducted in Ethiopia (12, 15). But studies conducted in Nepal and Somalia have reported an association between age and PTSD (36, 41). These discrepancies may be due to differences in methodology and population characteristics.

The study found that women had a greater risk of developing PTSD compared to men, which is consistent with previous research (32, 40, 42, 49, 57). This may be due to women having a lower threshold for psychotrauma, increasing their likelihood of developing PTSD (12, 47), or because they tend to react to stress in a more emotional and rumination-focused manner than males. Community-based studies conducted in England also demonstrate that women score higher on PTSD measures than men (61).

In comparison to other studies, the current study found that participants who live in urban areas had a 2.8 times greater likelihood of developing PTSD compared to those in rural areas. This finding is consistent with some previous research, which has suggested that urban living may be a risk factor for PTSD (36, 62). This may be due to rapid rural-to-urban migration, causing unplanned urbanization and more hardship in urban areas for this group of people.

Participants with no formal education in this study were found to have a higher odd of having PTSD, and this is similar to findings from a previous study in South Africa (52). Other studies have shown conflicting results regarding the association between PTSD and education (32, 41, 42, 53).

Marital status did not show a significant association with the development of PTSD among trauma patients in this study, consistent with previous studies conducted in Ethiopia and Somalia (36, 53). But these findings are inconsistent with studies conducted in Nigeria and Nepal (41, 59, 62). These differences may be attributed to factors other than marital status, such as the severity of trauma and the presence of social support networks, which may have a more significant impact on the development of PTSD. Methodological differences and population characteristics could also contribute to the discrepancies.

There was no significant association found between employment, income, and the presence of PTSD among trauma patients in this study, which is inconsistent with previous studies (41, 42, 53, 61). This might be due to the variation in sample size and methodology used.

Similarly, the negative association between social support and PTSD in this study aligns with findings from other studies. This may be because trauma patients who lack social support are more likely to experience negative psychological effects, leading to poorer mental health outcomes. Positive social support appears to enhance coping capacity in such circumstances (40–42, 57).

Individuals with depression were found to have a higher risk of having PTSD compared to those without depression, which is consistent with previous research (32, 40, 47). This could be due to depression increasing the risk of trauma exposure, impairing help-seeking behavior and effective coping strategies, or shared risk factors between depression and PTSD, such as poor social support or other stressful life events.

The association between exposure to multiple traumatic events and the risk of developing PTSD has also been reported in previous research (36, 47). This could be because when an individual is exposed to multiple traumatic events, they have a greater negative influence on mental health than a single, discrete trauma.

Overall, the findings of this study are generally consistent with previous research on the risk factors for mental illness among trauma patients. The study highlights the importance of addressing socioeconomic and psychosocial factors, such as education, income, employment status, and social support, in the management and treatment of mental health disorders among trauma patients.

## Limitations of the study

This study acknowledges limitations. While it offers valuable insights into the mental health of trauma outpatients, the inherent constraints of cross-sectional studies prevent causal inferences regarding the relationships between exposure and outcomes.

The study relied on self-reported measures (PHQ-9 and PCL-5) for identifying depression and PTSD, which may not align perfectly with clinical diagnostic criteria, potentially leading to an overestimation or underestimation of the prevalence of these disorders. Additionally, participants were asked to recall past events, introducing the risk of recall bias, and some sensitive traumas may not have been disclosed. Given that the research was conducted in institutional settings, the results may not be applicable to the broader population.

Despite these limitations, the study’s strengths include its comprehensive involvement of all public hospitals with trauma centers and high patient loads, maximizing representativeness. The use of updated standardized tools for measuring trauma, PTSD, and depression, along with close field supervision during data collection, helped mitigate missing data and reinforced the study’s reliability.

## Conclusion

The study revealed a high prevalence of depression and PTSD among trauma patients, with the most reported traumatic events being life-threatening illnesses, severe human suffering, transportation accidents, and physical assaults. Key factors associated with psychiatric morbidity included female gender, lower education levels, unemployment, low income, multiple traumatic exposures, and poor social support. These findings align with existing literature and underscore the urgent need for a comprehensive approach to mental health care for trauma patients in Ethiopia.

There is a clear necessity for structured mental health support systems in Ethiopian trauma clinics, particularly for marginalized groups such as women and low-income populations. Addressing the identified limitations and implementing context-specific interventions will significantly enhance the study’s contributions to the field. Future research should prioritize more representative sampling methods to capture the diversity of experiences across the country. Incorporating both self-reported measures and clinician-administered diagnostic assessments will provide a more nuanced understanding of the mental health burden and inform appropriate clinical interventions and support services.

## Data Availability

The original contributions presented in the study are included in the article/supplementary material. Further inquiries can be directed to the corresponding author.
